# Impact of Mitomycin-C-Induced Neutropenia after Hyperthermic Intraperitoneal Chemotherapy with Cytoreductive Surgery in Colorectal Cancer Patients with Peritoneal Carcinomatosis

**DOI:** 10.1245/s10434-021-10924-z

**Published:** 2021-10-19

**Authors:** Suk Jun Lee, Youngbae Jeon, Hae Won Lee, Jeonghyun Kang, Seung Hyuk Baik, Eun Jung Park

**Affiliations:** 1 Department of Surgery, Yonsei University College of Medicine, Seoul, Republic of Korea; 2 Department of Surgery, Gil Medical Center, Gachon University, Incheon, Republic of Korea; 3 Department of Surgery, Asan Medical Center, University of Ulsan College of Medicine, Seoul, Republic of Korea; 4 Division of Colon and Rectal Surgery, Department of Surgery, Gangnam Severance Hospital, Yonsei University College of Medicine, 211 Eonju-ro, Gangnam-gu, Seoul, Republic of Korea

## Abstract

**Background:**

Mitomycin-C (MMC) is the most commonly used chemotherapeutic agent for hyperthermic intraperitoneal chemotherapy (HIPEC) after cytoreductive surgery (CRS). However, MMC has a side effect of myelosuppression. This study aimed to evaluate the clinical manifestations and impact of MMC-induced neutropenia after CRS and HIPEC in colorectal cancer patients.

**Methods:**

A total of 124 colorectal cancer patients who underwent CRS with HIPEC between March 2015 and January 2019 were evaluated. Patients with malignancies of non-colorectal origin, hospital stay longer than 60 days, peritoneal cancer index > 30, and complete cytoreduction score > 2 were excluded. MMC 35 mg/m^2^ was administered for 90 min at 41–43 °C. The patients were divided into three groups: no neutropenia, mild neutropenia (grade 1–2), and severe neutropenia (grade 3–4).

**Results:**

In total, mild and severe neutropenia occurred in 30 (24.2%) and 48 (38.7%) patients, respectively. Age and body surface area were significantly different among the neutropenia groups. Severe neutropenia developed significantly earlier than mild neutropenia (6.9 days vs. 10.4 days, *p* < 0.001) and also lasted significantly longer (4.6 days vs. 2.5 days, *p* = 0.005). The rate of major postoperative complications was significantly higher in the severe neutropenia group than in the no and mild neutropenia groups (8.3% vs. 6.7% vs. 6.5%, *p* = 0.015)

**Conclusions:**

Severe neutropenia starts earlier and lasts longer than mild neutropenia after CRS and HIPEC using an MMC triple method. The higher rate of major postoperative complications in patients with severe neutropenia highlights the importance of postoperative management during the neutropenia period.

Hyperthermic intraperitoneal chemotherapy (HIPEC) after cytoreductive surgery (CRS) is an established effective method to treat colorectal cancer with peritoneal carcinomatosis.^1,2^ Although there have been advances in modern chemotherapy, CRS with HIPEC for reduction of the tumor burden and direct drug absorption into the peritoneum is expected to remain the mainstay of treatment as it overcomes the limitations of systemic chemotherapy for peritoneal metastases.^3,4^

HIPEC is based on the pharmacologic principles of passive diffusion, direct infiltration, and re-circulation of anticancer drugs, which are different from systemic chemotherapy.^5–7^ The peritoneal-plasma barrier enables increased drug absorption into tumors by maintaining a high concentration of anticancer drugs in the peritoneal space and decreasing plasma clearance. Given these different pharmacologic properties of HIPEC, the appropriate anticancer drugs are carefully selected.^5,8,9^

Historically, mitomycin-C (MMC) and oxaliplatin were widely used for HIPEC to treat colorectal cancer. An oxaliplatin-based regimen has been generally used as the standard regimen for HIPEC, although it has been recently switched to an MMC-based HIPEC in Europe based on the results of the French PRODIGE-7 trial.^10^ Thus, MMC is now the most commonly used chemotherapeutic in HIPEC for colorectal cancer. MMC is an antitumor antibiotic that inhibits DNA synthesis. It has a molecular weight of 334 Da, is water soluble, and has a heat-synergetic effect.^11,12^ MMC has a relatively longer half-life and higher peritoneal-plasma area under the curve (AUC) than oxaliplatin,^13^ thus making it a proper HIPEC chemotherapeutic agent to treat colorectal cancer patients. The randomized controlled trial by Verwaal et al. used MMC for HIPEC and reported better survival benefits than those with systemic chemotherapy in colorectal cancer patients with peritoneal metastasis.^1,2^

However, despite these benefits, MMC has adverse effects of delayed myelosuppression and nephrotoxicity.^12,14^ Particularly, severe neutropenia after CRS with HIPEC can be a life-threatening condition. Schnake et al. reported a 66% mortality rate for chemotherapy-induced neutropenia after HIPEC using MMC.^15^ However, despite the severe risks of this side effect, few studies have evaluated postoperative neutropenia after HIPEC using MMC. Therefore, this study aimed to investigate the incidence patterns and clinical manifestations of MMC-induced neutropenia after CRS with HIPEC and its impact in colorectal patients with peritoneal metastases.

## Methods

### Study Design and Population

This was a retrospective study of 305 colorectal cancer patients with synchronous or metachronous peritoneal metastases who underwent CRS with HIPEC at Gangnam Severance Hospital, Yonsei University College of Medicine, Seoul, South Korea between March 2015 and January 2019. Among them, patients who underwent surgeries for liver or lung metastases from colorectal cancer were included. The exclusion criteria were appendiceal neoplasm, pseudomyxoma peritonei, or non-colorectal primary malignancies; peritoneal cancer index (PCI) over 30; complete cytoreduction (CC) score of 3; and Eastern Cooperative Oncology Group (ECOG) performance status score > 2. Patients admitted for > 60 days postoperatively were also excluded to avoid the bias of neutropenia affected by septic conditions during long hospitalizations. Finally, 124 patients were evaluated. Data were collected prospectively and analyzed retrospectively.

This study was approved by the Institutional Review Board of our institution (No. 3-2020-0315) and was conducted according to the tenets of the Helsinki Declaration.

### Definition of Neutropenia

**Fig. 1:**
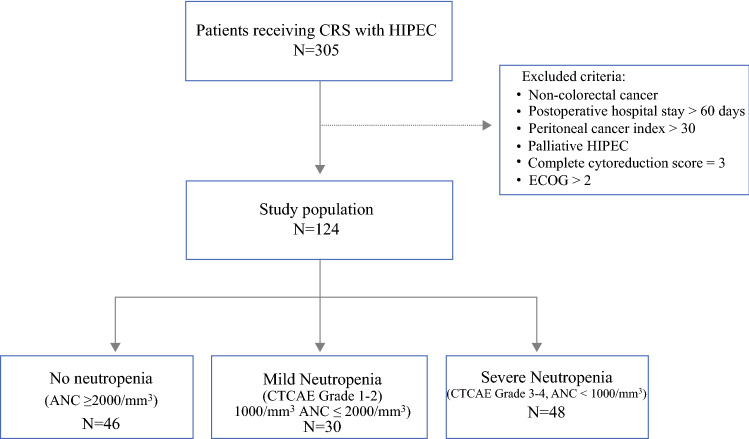
Patient selection flow chart (ANC,/mm^3^). *CRS*, cytoreductive surgery; *HIPEC*, hyperthermic intraperitoneal chemotherapy

Neutropenia was defined according to the National Cancer Institute’s Common Terminology Criteria for Adverse Events (CTCAE) grading of hematologic toxicity.^16^ The grade of neutropenia was categorized by absolute neutrophil count (ANC). The patients were divided into three groups: the no neutropenia, mild neutropenia, and severe neutropenia groups (Fig. 1). No neutropenia was defined as an ANC greater than our institutional lower limit of normal (LLN) of 2000/mm^3^. Mild neutropenia was defined as grade I–II neutropenia with an ANC of ≥ 1000 but < 2000/mm^3^. Meanwhile, severe neutropenia was defined as grade III–IV neutropenia with an ANC < 1000/mm^3^. Filgrastim (0.3 mg/vial), a granulocyte colony-stimulating factor (G-CSF), was administered subcutaneously once daily for severe neutropenia.

### Procedures of Cytoreductive Surgery and HIPEC

CRS was performed with surgically complete removal of gross tumors in the abdominal cavity. In the patients with synchronous colorectal cancer with peritoneal metastases, primary tumor resection and cytoreduction of peritoneal metastases were performed concurrently. Combined resections of oligometastases in the liver or lung were also performed. When diaphragmatic peritonectomy was performed, chest tube insertion was performed intraoperatively. After CRS, HIPEC was performed using MMC 35 mg/m^2^ at 41–43 ºC for 90 min. Following the HIPEC triple method, MMC 35 mg/m^2^ was mixed with 3 l of Dianeal® PD-2 1.5% peritoneal dialysis solution (Baxter, USA) and administered into the intraperitoneal cavity at 50% of the dose at the beginning of HIPEC, 25% of the dose at 30 min, and 25% of the dose at 60 min. All HIPEC procedures were performed with the open method using coliseum techniques. All procedures related with bowel anastomosis were performed after HIPEC.

### Evaluation Parameters

Hematologic blood tests to evaluate hemoglobin levels and leukocyte, platelet, and neutrophil counts were performed once a day from the operative day to discharge. Basic patient information such as sex, age, body mass index (BMI), history of abdominal operation, American Society of Anesthesiologists (ASA) score, and location of the primary cancer were recorded prospectively. Body surface area (BSA) was calculated by Mosteller formula in all patients. Intraoperative data such as PCI, CC scores, and operative procedures were evaluated. Operation time, the amount of intraoperative blood loss, and intraoperative transfusion were recorded postoperatively. The length of hospital stay was measured from the day of operation to discharge. Re-admission was defined as admission within 30 days after discharge. Postoperative complications were classified following the Clavien–Dindo classification.^17^ The occurrence patterns of neutropenia were evaluated postoperatively by measuring the ANC. The duration of neutropenia was measured from the date on which the ANC decreased below the LLN to the date on which it recovered to normal levels. The time of neutropenia onset, neutropenia duration, and frequency of G-CSF use were compared between patients with mild and severe neutropenia. A second-generation cephalosporin and metronidazole were used for 7 and 3 days postoperatively, respectively, after CRS followed by HIPEC. Extended antibiotic use as prophylaxis was not routinely implemented in patients who developed severe neutropenia. However, they were used in patients who had severe neutropenia with neutropenia fever or infectious conditions, after consulting infectious disease specialists.

### Statistical Analysis

Categorical variables were calculated using the chi-square test or Fisher’s exact test, whereas continuous variables were evaluated using the independent *t*-test or one-way analysis of variance among groups. Post-hoc analysis was conducted using the least significant different method comparing the three patient groups. All statistical analyses were conducted using IBM SPSS Statistics for Windows, version 25.0 (IBM Corp., Armonk, NY, USA). A *P*-value less than 0.05 was regarded to be statistically significant.

## Results

### Baseline Patient Characteristics

Postoperative neutropenia occurred in 78 (62.9%) patients; of them, 30 patients (24.2%) and 48 patients (38.7%) developed mild and severe neutropenia, respectively (Fig. 1). The severe neutropenia group was significantly older than the no or mild neutropenia group (*p* = 0.004). The preoperative BSA was significantly higher in the no neutropenia group than in the mild or severe neutropenia group (no vs mild neutropenia *p* = 0.044; no vs severe neutropenia, *p* = 0.034). Meanwhile, there was no significant among-group differences for sex, BMI, and previous histories of abdominal operations. There were also no significant differences in ASA and ECOG performance status among the groups. Primary colorectal cancer resection was performed before CRS with HIPEC in 58.3%, 70.0%, and 56.5% of the patients in the severe, mild, and no neutropenia groups, respectively. In all groups, the primary tumor was most commonly located in the sigmoid colon, followed by the ascending colon. Although there were six patients who had synchronous liver metastasis in the no neutropenia group, there was no significant difference in the synchronous metastatic sites among the groups. Patients over 75% received chemotherapy before CRS with HIPEC but had no significant difference among groups. The preoperative leukocyte and platelet counts and the carcinoembryonic antigen level were also not significantly different (Table 1).

**Table 1:** Patient characteristics by neutropenia groups

Variables	No neutropenia N=46	Mild neutropenia N=30	Severe neutropenia N=48	*P*-value
Sex				0.128
Male	24 (52.2)	9 (30.0)	18 (37.5)	
Female	22 (47.8)	21 (70.0)	30 (62.5)	
Age, years	50.7±14.4 (20–80)	53.3±12.5 (24–75)	59.4±10.6 (20–80)	0.004 (No vs Mild *p*=0.369, No vs Severe *p*=0.001, Mild vs severe *p*=0.041)
BMI, kg/m^2^	23.7±4.0 (16.1–33.0)	22.6±3.80 (15.4–31.0)	23.1±2.90 (15.4–33.0)	0.388
BSA, m^2^	1.7±0.2 (1.3–2.1)	1.6±0.2 (1.3–2.1)	1.6±0.2 (1.1–2.0)	0.052 (No vs Mild *p*=0.044, No vs Severe *p*=0.034 Mild vs. severe p=0.880)
History of abdomen operation	29 (63.0)	22 (73.3)	33 (68.7)	0.632
Past primary tumor resection	26 (56.5)	21 (70.0)	28 (58.3)	0.465
*ASA*				0.067
1	4 (8.7)	6 (20.0)	3 (6.3)	
2	27 (58.7)	11 (36.7)	33 (68.7)	
3	15 (32.6)	13 (43.3)	12 (25.0)	
ECOG performance score				0.109
0	16 (34.8)	13 (43.3)	11 (22.9)	
1	24 (52.2)	17 (56.7)	29 (60.4)	
2	6 (13.0)	0 (0.0)	8 (16.7)	
Location of primary cancer				0.028 (No vs. Mild *p*=0.299, No vs. Severe *p*=0.157, Mild vs. Severe *p*=0.006)
Ascending colon	8 (17.4)	4 (13.3)	16 (33.3)	
Cecum	2 (4.3)	0 (0.0)	5 (10.3)	
Transverse colon	3 (6.5)	4 (13.3)	1 (2.1)	
Descending colon	1 (2.2)	3 (10.0)	2 (4.2)	
Sigmoid colon	20 (43,5)	11 (36.7)	20 (41.7)	
Rectosigmoid junction	6 (13.0)	1 (3.3)	2 (4.2)	
Rectum	6 (13.0)	7 (23.3)	2 (4.2)	
Synchronous metastasis Liver Lung Ovary/adnexa	6 (13.0) 6 0 0	1 (3.3) 1 0 0	2 (4.2) 2 0 0	0.247
Previous chemotherapy				0.875
*No*	10 (21.7)	8 (26.7)	12 (25.0)	
*Yes*	36 (78.3)	22 (73.3)	36 (75.0)	
1^st^ line	21 (58.4)	15 (68.3)	26 (72.2)	
2^nd^ line	12 (33.3)	4 (18.2)	6 (16.7)	
≥3^rd^ line	3 (8.3)	3 (4.5)	4 (11.1)	
Preoperative WBC, 10^3^/µl	6231.5±2255.4 (2480–12100)	5928.0±2005.3 (2820–11390)	6338.5±2145.3 (2360–11600)	0.710
Preoperative platelet, 10^3^/µl	251.1±84.2 (81–527)	254.4±76.2 (130–425)	259.8±107.9 (68–568)	0.900
Preoperative CEA, ng/ml	28.8±71.9 (0.4–408.6)	46.0±89.1 (1.2–386.9)	27.1±52.0 (0.8–258.4)	0.468

*ANC*, Absolute neutrophil count; *BMI*, body mass index; *BSA*, body surface area; *ASA*, American Society of Anesthesiologists; *ECOG*, Eastern Cooperative Oncology Group; *WBC*, white blood cell; *CEA*, carcinoembryonic antigen.

### Comparison of Perioperative Outcomes among the Neutropenia Groups

Both the PCI and CC scores did not differ significantly among the groups (Table 2). Although 18.8% of the patients in the severe neutropenia group had a PCI over 20, there was no significant difference in the PCI and occurrence of neutropenia (*p* = 0.320). Overall, 93.5%, 100%, and 97.9% of the patients in the no, mild, and severe neutropenia groups had a CC score of 0/1, respectively. There were no significant differences in the rates of primary tumor resection and stoma formation among the groups. In total, 52.2%, 40.0%, and 52.1% of patients in the no, mild, and severe neutropenia groups underwent combined resection for metastatic lesions, respectively (*p* = 0.512). Splenectomy was performed in 13.0%, 10.0%, and 6.3% of patients in the corresponding groups, respectively, with no significant difference (*p* = 0.545). There was also no significant difference in operative time or intraoperative transfusion. The length of hospital stay also did not differ significantly: 15.3 days, 17.6 days, and 18.0 days in the no, mild, and severe neutropenia groups, respectively (*p* = 0.246).

**Table 2:** Comparison for the perioperative outcomes

Variables	No neutropenia N=46	Mild neutropenia N=30	Severe neutropenia N=48	*P*-value
*Peritoneal cancer index (PCI)*				0.320
PCI < 10	25 (54.3)	22 (73.3)	24 (50.0)	
10≤ PCI < 20	14 (30.4)	6 (20.0)	15 (31.2)	
20≤ PCI < 30	7 (15.2)	2 (6.7)	9 (18.8)	
Complete cytoreduction (CC)				0.728
CC-0	39 (84.8)	27 (90.0)	43 (89.6)	
CC-1	4 (8.7)	3 (10.0)	4 (8.3)	
CC-2	3 (6.5)	0 (0.0)	1 (2.1)	
*Resection of primary tumor*				0.465
No	26 (56.5)	21 (70.0)	28 (58.3)	
Yes	20 (43.5)	9 (30.0)	20 (41.7)	
Rt. hemicolectomy	5 (10.9)	2 (6.7)	9 (18.8)	
Lt. hemicolectomy	2 (4.3)	0 (0.0)	1 (2.1)	
Anterior resection	3 (6.5)	0 (0.0)	4 (8.3)	
Low anterior resection	9 (19.6)	7 (23.3)	5 (10.4)	
Hartmann’s operation	0 (0.0)	0 (0.0)	1 (2.1)	
Total colectomy	1 (2.2)	0 (0.0)	0 (0.0)	
*Combined organ resection*				0.512
No	22 (47.8)	18 (60.0)	23 (47.9)	
Yes*	24 (52.2)	12 (40.0)	25 (52.1)	
Liver resection	7 (15.2)	3 (10.0)	11 (22.9)	
Intraoperative RFA	2 (4.3)	1 (3.3)	1 (2.1)	
Lung resection	0 (0.0)	0 (0.0)	0 (0.0)	
Ureteroneocystostomy	5 (10.9)	3 (10.0)	1 (2.1)	
Ureteral stent insertion	5 (10.9)	2 (6.7)	3 (6.3)	
Distal pancreatectomy	1 (2.2)	1 (3.3)	0 (0.0)	
Cholecystctomy	23 (50.0)	17 (56.7)	23 (47.9)	
TAH with BSO	10 (21.7)	10 (33.3)	13 (27.1)	
Splenectomy	6 (13.0)	3 (10.0)	3 (6.3)	
Seminal vesiculectomy	2 (4.3)	1 (3.3)	0 (0.0)	
*Stoma formation*				0.170
Jejunostomy or ileostomy	2 (4.3)	5 (16.7)	8 (16.7)	
Colostomy	9 (19.6)	3 (10.0)	4 (8.3)	
Operation time, h	8.9±3.3 (2.7–15.8)	8.5±2.8 (4.2–15.0)	9.2±3.4 (3.4–21.7)	0.626
Intraoperative transfusion	18 (39.1)	9 (30.0)	19 (39.6)	0.652
Length of hospital stay, days	15.3±8.7 (8–42)	17.6±9.5 (9–54)	18.0±6.6 (10–40)	0.246

*Number of patients who received combined resection. *TAH*, Total abdominal hysterectomy; *BSO*, bilateral salpingo-oophorectomy.

### Time Profiles and Clinical Manifestations of MMC-Induced Neutropenia

**Fig. 2:**
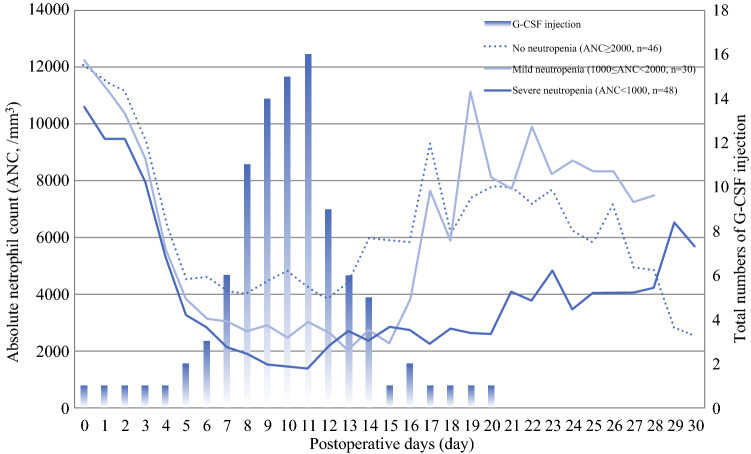
Time profiles of mitomycin-C induced neutropenia after CRS with HIPEC

All three groups tended to show a postoperative gradual decrease of ANC after CRS and HIPEC. Although the ANC was recovered by postoperative day 11 in most patients, the onset of neutropenia was different between the mild and severe neutropenia groups (Fig. 2). Severe neutropenia occurred significantly earlier than mild neutropenia (6.9 days vs. 10.4 days, *p* < 0.001). Severe neutropenia also lasted longer than mild neutropenia (4.6 days vs. 2.5 days, *p* = 0.005). The ANC of the severe neutropenia group reached a nadir phase within 10.9 postoperative days, whereas that of the mild neutropenia group was at 11.5 postoperative days (*p* = 0.532). Particularly, there were four patients who had ANC less than 100/mm^3^ during the severe neutropenia period. G-CSF injection was initiated in patients with severe neutropenia at postoperative 8.3 days and used for an average of 2.5 days. G-CSF administration peaked at postoperative day 11 and was reduced subsequently as the ANC recovered. No patient in the mild neutropenia group developed complications, but 25% of those in the severe neutropenia had postoperative complications that required re-operations owing to rectal fistula and anastomotic bleeding (Table 3).

**Table 3:** Comparison of clinical characteristics for MMC-HIPEC-induced neutropenia between mild and severe neutropenia groups

Variables	Mild neutropenia N=30	Severe neutropenia N=48	*P*-value
Postoperative days to begin neutropenia*, day	10.4±3.7 (4–16)	6.9±2.8(0–14)	<0.001
Duration of neutropenia*, day	2.5±1.9 (1–8)	4.6±2.7 (1–11)	0.005
Postoperative day at the lowest ANC count*, day	11.5±3.8 (4–18)	10.9±3.5 (0–23)	0.532
Postoperative days to recover neutropenia *, day	11.8±4.1 (7–20)	11.1±3.7 (1–17)	0.554
Postoperative day to begin G-CSF injection*, day	–	8.3±4.5 (0–16)	–
Duration of use of G-CSF*, day	–	2.5±2.8 (1–6)	–
*Complications during neutropenia period‡*			–
No	30 (100.0%)	36 (75.0%)	
Yes	0 (0.0%)	12 (25.0%)	
Re-operation during neutropenia period‡	0 (0.0%)	2 (4.2%) Rectal fistula (1) Anastomosis site bleeding (1)	–

*ANC*, Absolute neutrophil count; *G-CSF*, granulocyte colony-stimulating factor

*days; ANC < 1500; †/µl; ‡, N(%)

### Comparison of Postoperative Outcomes and Complications

The rates of postoperative complications after CRS with HIPEC were significantly high in the severe neutropenia group (*p* = 0.015). In a post-hoc analysis, the rates of major postoperative complications were higher in the severe neutropenia group than in the no and mild neutropenia groups (8.3% vs. 6.7% vs. 6.5%). The rates of grade I–II postoperative complications were higher in the severe neutropenia group than in the no and mild neutropenia groups (81.3% vs. 60.0% vs. 54.3%). Meanwhile, there was no significant difference in re-admission rates. The starting period of both grade I–II and grade ≥ III postoperative complications had no significant effect on neutropenia development (Table 4).

**Table 4:** Comparison of postoperative complications by neutropenia groups

Variables	No neutropenia N=46	Mild neutropenia N=30	Severe neutropenia N=48	*P*-value
*Postoperative complications*^*†*^				0.015 (No vs. Mild *p*=0.868, No vs. Severe *p*=0.004 Mild vs. Severe *p*=0.044)
Grade I-II	25 (54.3)	18 (60.0)	39 (81.3)	
Grade III-V	3 (6.5)	2 (6.7)	4 (8.3)	
Grade IIIa	0 (0.0)	1 (3.35) Pleural effusion	0 (0.0)	
Grade IIIb	2 (4.3) Ischemic colitis (1) Rectal fistula (1)	1 (3.35) Wound evisceration	2 (4.15) Anastomotic bleeding (1) Rectal fistula (1)	
Grade IV	1 (2.2) Anastomosis leakage		2 (4.15) Pneumonia (1) Stroke (1)	
Grade V	0 (0.0)	0 (0.0)	0 (0.0)	
Re-admission	19 (41.3)	6 (20.0)	11 (22.9)	0.066
Complication-related	8 (17.4)	3 (10.0)	3 (6.3)	
Non-complication related	11 (23.9)	3 (10.0)	8 (16.7)	
*Initiation of complications in the postoperative period, days*				
Grade I-II	6.3±6.2 (2–25)	5.3±4.6 (0–17)	6.4±4.6 (0–24)	0.712
Grade ≥ III	15.0±8.8 (7–27)	8.5±2.1 (7–10)	13.0±7.7 (2–23)	0.636

†, According to Clavien-Dindo classification.

## Discussion

Data on postoperative neutropenia after HIPEC using MMC are rare. In this study, severe and mild neutropenia occurred in 38.7% and 24.2% of the patients who underwent CRS with HIPEC using the MMC triple method, respectively. Particularly, severe neutropenia developed early at postoperative day 7 and lasted longer than mild neutropenia. The rates of postoperative complications were higher in patients with severe neutropenia than in those with no or mild neutropenia.

MMC is the most widely used chemotherapeutic agent in HIPEC for colorectal cancer with peritoneal metastases owing to its antitumor effect and high rate of AUC ratio between the concentration of peritoneal fluid and plasma.^5,12^ However, MMC has a side effect of delayed myelosuppression that occurs in both systemic and intraperitoneal chemotherapies.^7,12^ Other adverse effects include renal toxicity, diarrhea, and anorexia. The surgical stress after CRS and postoperative MMC-induced myelosuppression after HIPEC are important concerns because they delay postoperative recovery and increase the risk of postoperative complications.

MMC-induced severe neutropenia after HIPEC occurs in 20–40% of patients.^14,18–20^ In this study, the overall incidence of neutropenia after CRS followed by HIPEC was 62.9% and that of severe neutropenia was 38.7%. To analyze the detailed clinical manifestations during the neutropenia period after HIPEC, we divided the patients into mild and severe neutropenia according to the CTCAE hematologic toxicity grade. Notably, most patients in all groups showed similar patterns with serial decrease of neutrophil count postoperatively after CRS with HIPEC. However, MMC-induced neutropenia developed earlier and the recovery time was longer in patients with severe neutropenia than in those with mild neutropenia. Severe neutropenia developed by postoperative day 7 and lasted for 4.6 days. These results suggest that the risk of immunosuppression-related infectious complications should be closely monitored starting from 1 week after CRS with HIPEC using MMC. In our institution, serologic examination and use of prophylactic antibiotics until postoperative days 7–10 are used to prevent myelosuppression-related complications after CRS with HIPEC. It is crucial that serial serologic markers such as changes in hemoglobin, neutrophils, and platelet counts are measured to detect the severity of immunosuppression postoperatively.

Sex, BSA, and intraoperative concentration of MMC are established predisposing factors for neutropenia after CRS with HIPEC.^14,18,19^ However, the clinical factors affecting MMC-induced neutropenia after HIPEC are yet to be identified because most studies have been retrospective in design and had small sample sizes. Lambert et al. reported that female sex and MMC dose standardized for BSA increased the risk of neutropenia after CRS with HIPEC in 117 patients receiving MMC concentrations of 7.5–10 µg/ml for 90 min.^18^ Meanwhile, Kemmel et al. evaluated the clinical risk factors of neutropenia according to the pharmacokinetics of MMC in 45 patients who underwent HIPEC with MMC (0.8 mg/kg for 90 min).^14^ They found that the MMC plasma concentration at 30 min after HIPEC was significantly higher in patients with neutropenia than in those with no neutropenia.

The current study found significant differences in age and BSA among the three groups. The difference in BSA reflects the peritoneal surface in contact directly with anticancer drugs and correlates with the drug absorption rate during HIPEC. Although both the PCI and combined organ resection did not affect postoperative neutropenia after CRS with HIPEC, the results suggest that preoperative BSA should be carefully considered when calculating the MMC dose for HIPEC. Given that Asian patients tend to have relatively lower BSA and BMI than Western patients, more individualized standards to determine the MMC dose are required. In the randomized controlled study by Verwaal et al. that used the same HIPEC triple method as in our study, grade 3–4 leukopenia and thrombocytopenia occurred in 17% and 4% of the patients, respectively.^1^ However, in the current study of Korean patients, 38.7% developed severe neutropenia. As such, a detailed guideline to adjust the MMC dose according to a patient’s BSA might be required.

MMC-induced neutropenia after CRS with HIPEC is a major concern because it increases the risk of postoperative complications from bone marrow suppression. The major morbidity rates after CRS with HIPEC range from 20% to 50%.^21–24^ Sugarbaker et al. reported that the most common grade IV complications were hematologic problems (28%) and gastrointestinal complications (26%).^22^ In the current study, the rates of major complications were 8.3% in the severe neutropenia group and 6.7% in the mild neutropenia group. The major complications in the severe neutropenia group were related to anastomotic bleeding and rectal fistula. In addition, the rates of postoperative grade I–II complications were higher in the severe neutropenia than in the no or mild neutropenia groups. Surgical stress and systemic inflammatory response are higher in CRS procedures combined with HIPEC than in primary tumor resection alone. In addition, intraoperative hyperthermia during HIPEC increases the systemic inflammatory response. The levels of interleukin (IL)-18, IL- 6, and tumor necrosis factor-α are increased in patients undergoing CRS with HIPEC.^25,26^ Surgical stress and immunosuppression from the hematologic toxicities of MMC might further delay wound healing and postoperative recovery.^27^ Considering that postoperative complications worsen oncologic outcomes^24^, the MMC dose should be optimized, and postoperative complications should be prevented during the neutropenia period after CRS with HIPEC.

This study is meaningful in that it demonstrates the time-dependent serologic patterns of MMC-induced neutropenia and its clinical manifestations after CRS with HIPEC with the triple method. Particularly, the clinical course of neutropenia found in this study might be helpful to detect and prevent severe neutropenia during the postoperative period. However, this study also has the limitations of a retrospective design, small sample size, and unavoidable selection bias. Prospective clinical trials are needed to identify reliable predictors of MMC-induced neutropenia and establish an appropriate MMC dose stratified by the risk of neutropenia.

In conclusion, severe neutropenia begins earlier and lasts longer than mild neutropenia in CRS with HIPEC using the MMC triple method. Severe neutropenia increases the risk of major postoperative complications, and thus it is crucial to pay attention to postoperative management during the neutropenia period. Postoperative neutropenia and the optimal MMC dose for HIPEC should be accurately assessed to lower the risk of future postoperative complications.
